# Genome–scale approach to study the genetic relatedness among *Brucella melitensis* strains

**DOI:** 10.1371/journal.pone.0229863

**Published:** 2020-03-09

**Authors:** Ana Pelerito, Alexandra Nunes, Maria Sofia Núncio, João Paulo Gomes

**Affiliations:** 1 Department of Infectious Diseases, Emergency Response and Biopreparedness Unit, National Institute of Health, Lisbon, Portugal; 2 Department of Infectious Diseases, Bioinformatics Unit, National Institute of Health (INSA), Lisbon, Portugal; East Carolina University Brody School of Medicine, UNITED STATES

## Abstract

Brucellosis is an important zoonotic disease that affects both humans and animals. To date, laboratory surveillance is still essentially based on the traditional MLVA-16 methodology and the associated epidemiological information is frequently scarce. Our goal was to contribute to the improvement of *Brucella* spp. surveillance through the implementation of a whole genome sequencing (WGS) approach. We created a curated ready-to-use species-specific wgMLST scheme enrolling a panel of 2656 targets (http://doi.org/10.5281/zenodo.3575026) and used this schema to perform a retrospective analysis of the genetic relatedness among *B*. *melitensis* strains causing human infection in Portugal (a country where brucellosis is an endemic disease) from 2010 to 2018. The strains showed a phylogenetic clustering within genotype II (25 out of 36) and IV (4 out of 36), and shared clades with strains isolated from countries with which Portugal has intense food trading, tourism and similar eating habits, such as Spain, Italy and Greece. In addition, our results point to the identification of strong associations between *B*. *melitensis* strains, likely underlying missed “outbreaks” as 22 out of the 36 strains showed genetic linkage with others. In fact, the applied gene-by-gene approach grouped these strains into six genetic clusters each one containing putative epidemiological links. Nevertheless, more studies will be needed in order to define the appropriate range of cut-offs (probable non-static cut-offs) that best illustrate the association between genetic linkage and epidemiological information and may serve as alerts for the health authorities. The release of this freely available and scalable schema contributes to the required technological transition for laboratorial surveillance of brucellosis and will facilitate the assessment of ongoing and future outbreaks in order to prevent the transmission spread.

## Introduction

Brucellosis, a disease caused by *Brucella* spp., is one of the world’s most widespread zoonoses, with estimated 500 000 new cases annually, and it is the leading cause of economic losses in the production of domestic ruminants [1]. Although *Brucella* can infect multiple hosts, the frequent sources of human infections are farm animals such as cattle, sheep, goats and pigs. Humans can contract the disease by contact with infected animals or their products, with unpasteurized milk being the most common source of brucellosis in urban populations [2]. *Brucella* is a Gram-negative unsporulated and uncapsulated short bacillus that behaves as a facultative intracellular pathogen [3]. The genus *Brucella* encloses 12 species (although some of them are yet to be formally recognized by the International Taxonomy Committee of Brucellosis), designated based on differences in pathogenicity and host preference such as *B*. *melitensis* (goats and sheep), *B*. *abortus* (cattle), and *B*. *suis* (swine). *B*. *melitensis* is the most frequent agent of brucellosis in humans, and it leads to the most severe manifestations of the disease such as undulant fever, joint pain, arthritis, endocarditis and meningitis [4–6]. The *Brucella* genome contains two circular chromosomes of approximately 2.1 and 1.2Mb, and both share similar GC content, a similar proportion of coding regions and equivalent housekeeping gene distribution [7,8]. Also, three way genome comparisons of *B*. *suis*, *B*.*melitensis* and *B*. *abortus* sequences, revealed that the majority (>90%) of annotated genes shared 98–100% sequence identity and fewer than 100 genes were identified in only one or two of the three genomes [9]. Although prophages and insertion sequences have been reported [10–14], species from *Brucella* genus are nonetheless monomorphic pathogens [15].

The analysis of full genome sequences of the different species (and biovars) is of crucial importance, not only to disclose the genetic basis of host preference and virulence differences, but also for molecular surveillance purposes. Nevertheless, whole genome comparisons and phylogenetic analysis of *Brucella* have only been done on a limited scale. Efficient and reliable surveillance programs are essential for detection and control of outbreaks and largely depend on the timely collection and access to epidemiological data and the need of cooperation between different health sectors (i.e., human and veterinary) through the exchange of microbiological and associated metadata. In addition, complete epidemiological investigations rely on the availability of standardized and effective molecular typing methods and analysis tools that allow the public health laboratories to identify and trace an outbreak back to its source. Molecular epidemiological studies provide information about genetic grounds and origin of bacterial isolates, but such trace back studies in *Brucella* species can be challenging as genomes are generally quite conserved. With the technological advances and decreased cost of whole genome sequencing, new methods of pathogen typing, including gene-by-gene comparison using core genome multilocus sequence typing (cgMLST), as well as single-nucleotide polymorphism (SNP) calling based on a reference sequence analysis, are considered to be a suitable and more informative replacement of the gold standard typing schemes [16]. Although the SNP-based analysis may constitute a better option for phylogenetic analyses of conserved genomes (because this approach covers the entire genome, including the intergenic regions) [17], very recently, efforts to develop cgMLST schemes for *Brucella* have been done [16, 17]. One of these schemes involves 2704 genes and is based on a pay-per-use platform [17], whereas the other involves a strikingly low number of genes (n = 164) for differentiating purposes [16].

In Portugal, human brucellosis is a reportable disease and is among the three most frequent zoonosis [18]. This country has a herding tradition, with a high number of people keeping animals at farmhouses countrywide and with a long tradition of cheese production. Both Portuguese reference institutes for human and veterinary diagnosis of brucellosis using MLVA-16 (i.e., Multiple-Locus Variable number tandem repeat Analysis based on 16 loci) methodology as a typing technique in epidemiological studies [19], which is an important tool for molecular epidemiological surveillance. However, there is a lack of communication between human and animal health authorities and the epidemiological link is rarely established. Considering this and the need for a technological transition for surveillance purposes, we developed a wgMLST schema to perform a retrospective analysis of the genetic relatedness among *B*. *melitensis* strains causing human infections in Portugal. Ultimately, we aimed at identifying potential transmission links that have been missed with the currently implemented surveillance system. This study was based on the collection of *B*. *melitensis* strains held by the reference laboratory for human Brucellosis at the Portuguese National Institute of Health, which receives all human isolates of *B*. *melitensis*.

## Materials and methods

### Samples

This study enrolled, all *B*. *melitensis* strains that were sent to the reference laboratory for human Brucellosis at the Portuguese National Institute of Health during the last nine years, comprising 37 isolates. Genotyping and demographic data are summarized in Table 1. For genomic comparative purposes, it also included 18 strains isolated in Spain, Germany, Hungary and Belgium, which were kindly provided to our lab and that were subjected to all laboratory procedures and analysis (described below). For bioinformatics analysis, all *B*. *melitensis* genome sequences available at NCBI until January 2019 (n = 217) were also included yielding a total of 272 *B*. *melitensis* genomes to be analyzed.

**Table 1:** *Brucella melitensis* strains, data of origin, host and year.

Strain	Geographic Region	Host	Year
1P	Unknown	Human	2010
35P	Vila Real	Human	2012
36P	Vila Real	Human	2012
38P	Maia	Human	2012
40P	Vila Real	Human	2012
41P	Vila Real	Human	2012
43P	Vila Nova de Gaia	Human	2012
44P	Unknown	Human	2012
66P	Torres Novas	Human	2012
147P	Cabeceira de Basto	Human	2013
153P	Cabeceira de Basto	Human	2014
165P	Unknown	Human	2011
166P	Seixo de Ansiães	Human	2011
167P	Vila Franca de Xira	Human	2011
168P	Lourosa	Human	2011
169P	Unknown	Human	2014
177P	Unknown	Human	2014
179P	Baião	Human	2014
180P	Baião	Human	2014
184P	Baião	Human	2014
194P	Lisboa	Human	2015
198P	Lisboa	Human	2015
199P	Loures	Human	2015
200P	Pontinha	Human	2015
209P	Évora	Human	2015
228P	Caldas da Rainha	Human	2016
237P	Coimbra	Human	2016
258P	Vila Nova de Gaia	Human	2016
261P	Unknown	Human	2016
20Pa	Frei Rodrigo	Goat	2002
357Pa	Mafra	Sheep	2004
782Pa	Caldas da Rainha	Goat	2007
804Pa	Fundão	Bovine	2008
463Pa	Vila Viçosa	Sheep	2005
47Pa	Penamacor	Sheep	2001
770Pa	Vila do Conde	Sheep	2007
918Pa	Unknown	Goat	2011

All samples were handled in a BLS-3 biocontainment laboratory at the Portuguese National Institute of Health. *Brucella* isolates were cultured on blood agar for 3 to 5 days at 37° C under 5% CO2 and total DNA was extracted from fresh cultures on the NucliSens easyMAG platform (Biomerieux), according to the manufacturer’s instructions. All isolates had previously been confirmed as *Brucella* spp. by real-time PCR detecting the *Brucella* specific gene *IS711*, *BME* and *Brab* [20]. For simplification purposes, all *Brucella* isolates from the Portuguese collection that are enrolled in this study are designated with the prefix “PT” throughout the text.

### Antimicrobial susceptibility

All isolates were tested for antibiotic resistance to rifampicin (RIF), doxycycline (DOX), streptomycin (STR), gentamicin (GEN), by E- test^®^ (biomerieux, Portugal) according to Clinical and Laboratory Standards Institute (CLSI) guidelines for potential agents of bioterrorism. Briefly, a suspension of bacteria adjusted to 0.5 McFarland units was inoculated on Mueller—Hinton plates supplemented with 5% sheep blood and the gradient strips applied. The plates were incubated at 35 °C±2 °C with 5% CO2 for 48 h before reading. MIC values were interpreted in accordance with the CLSI guidelines [21]. The following breakpoints for susceptibilities were used: GEN≤4, STR≤16, DOX≤1. For RIF, CLSI interpretation of *Haemophilus infuenzae* (fastidious bacteria) was used: S≤1, I = 2, R≥4. Quality control assays were performed with *Escherichia coli* ATCC #25922 and *Streptococcus pneumoniae* ATCC #49619.

### Whole genome sequencing (WGS)

For WGS, high-quality DNA samples (quantified using Qubit, ThermoFisher) were subjected to dual-indexed Nextera XT Illumina library preparation, prior to cluster generation and paired-end sequencing (2×250bp) on a MiSeq Illumina platform (Illumina Inc.) available at the Portuguese NIH, according to the manufacturer’s instructions). All genomes were *de novo* assembled using the INNUca v3.1 pipeline (https://github.com/B-UMMI/INNUca), which consists of several integrated modules for reads QA/QC, *de novo* assembly and post-assembly optimization steps. Briefly, after reads’ quality analysis (FastQC v0.11.5 - http://www.bioinformatics.babraham.ac.uk/projects/fastqc/) and cleaning (Trimmomatic v0.36) [22], genomes were assembled with SPAdes 3.10 [23] and subsequently improved using Pilon v1.18 [24].

### Implementation of a wgMLST schema for *B*. *melitensis*

We created a wgMLST schema for *B*. *melitensis* with chewBBACA v2.0.11 suite (https://github.com/B-UMMI/chewBBACA) [24] (CreateSchema module; default settings), using all complete genomes of *B*. *melitensis* available at NCBI (until January 2019) and a training file generated by Prodigal v2.6.3 from the *B*. *melitensis* 16M reference genome (RefSeq Accession NC_003317 and NC_003318). To curate the schema, allele calling was performed on all complete genomes with default parameters using a BLAST Score Ratio (BSR) threshold of 0.6 in order to remove paralogous loci. A cgMLST schema was also extracted and allele calling was performed for all genomes of *B*. *melitensis* available at NCBI until January 2019 (that include 60 complete and 157 draft genomes) as well as for the 55 assemblies (that include sequences of 37 PT strains) performed in our lab, in order to discard genomes yielding less than 95% of called loci. To validate the wgMLST schema, allele calling was performed for the remaining assemblies. The impact of genome quality on allele call was evaluated (Test Genome Quality module) using a maximum number of interactions (-n) of 13 and exclusion thresholds from 0 to a maximum (-t) of 300 with increasing -s values of 5. Considering that the number of present loci varied with the inclusion or exclusion of specific genomes, a threshold of 25 was used to select genomes that allow a good discriminatory power for the wgMLST schema creation. The quality of the loci panel composing the wgMLST have been assessed using the Schema Evaluation module with default parameters. Basically, loci with high length variability, and annotated as “non-informative paralogous hit (NIPH/NIPHEM)” or “Allele larger/Smaller than length mode (ALM/ASM)” by the chewBBACA Alelle Calling engine in more than 1% of the *B*. *melitensis* genomes were removed in order to curate the wgMLST schema. Finally, exact and inferred matches were used to construct an allelic profile matrix, where the other allelic classifications (see https://github.com/B-UMMI/chewBBACA/wiki) were assumed as “missing” loci. The ready-to-use wgMLST schema for *B*. *melitensis* scheme enrolling a panel of 2656 loci is available at http://doi.org/10.5281/zenodo.3575026.

### Study of genetic relatedness among *B*. *melitensis* strains isolated in Portugal

In a second approach, minimum spanning trees (MST) were constructed solely for all PT strains taking advantage of goeBURST algorithm [24] implemented in the PHYLOViZ online web-based tool [25], based on 100% shared loci between all strains (corresponding to 2191 loci for the set of the PT strains). A hierarchical clustering tree were also generated using PHYLOViZ desktop 2.0 (http://www.phyloviz.net/) with distances among strains estimated with Hamming Distance metrics via the single-linkage method. In order to increase the resolution power for cluster analysis within the Portuguese strains, we used PHYLOViZ online 2.0 Beta version (http://online2.phyloviz.net), which allows maximizing the shared genome in a dynamic manner, i.e., for each sub-set of strains under comparison, the maximum number of shared loci between them is automatically used for tree construction. All allelic distance thresholds used during cluster investigation were expressed as percentages of allele differences (AD), expressed as the number of allelic differences over the total number of shared loci under comparison. To explore strain sub-sets among our 37 PT strains, a conservative step-by-step approach was performed by applying allelic distance cut-offs ranging from 1 to 0.1% to the initial MST, based on previously described data for cluster investigation in gene-by-gene based surveillance [26].

### Core-genome single nucleotide variant (SNV)-based analysis

Paralleling the wgMLST-based approach, a core-genome SNV-based analysis was also performed to evaluate the genetic relatedness among all Portuguese *B*. *melitensis* strains as it is a methodology largely used worldwide. Briefly, through a reference-based mapping strategy using Snippy4.1.0 software (https://github.com/tseemann/snippy), quality improved reads (after Trimmomatic processing as described above) were individually mapped against a representative draft assembled genome (Bm-147P was used as mapping reference for all 36 PT strains as well as for genotype IV cluster, while Bm-167P was used as mapping reference for genotype II cluster). SNV calling was performed on variant sites that filled the following criteria: *i)* minimum proportion of reads differing from the reference of 90%; *ii)* minimum mapping quality of 20; and *iii)* minimum number of reads covering the variant position >10. Core-SNPs were extracted using Snippy’s core module (snippy-core) ensuring that all genomes reached at least 99% of aligned bases with the reference. MEGA7 software (http://www.megasoftware.net) [27] was applied to calculate matrices of nucleotide distances and perform phylogenetic reconstructions over the obtained core-genome SNV alignment by using the Neighbor-Joining method [28] with the Maximum Composite Likelihood model to compute genetic distances [29] and bootstrapping (1000 replicates) [30].

### *In silico* Multilocus Sequence Typing (MLST) analysis

For comparative purposes, the traditional MLST 9-locus and 21-locus schemes (namely, MLST-9 [31] and MLST-21 [32] were also performed. For each MLST scheme, the *in silico* allele profile and corresponding sequence type (ST) were retrieved for each PT strain from PubMLST.org/brucella database (http://pubmlst.org/brucella/). MSTs were constructed using goeBURST algorithm [33] implemented in the PHYLOViZ online web-based tool [25].

## Results

All *Brucella* isolates were identified as *B*. *melitensis* by real-time PCR. The obtained MIC values for all tested antibiotics are shown in S2 Table. All isolates were susceptible to doxycycline, streptomycin and gentamicin. However, the MIC values for rifampicin ranged from 0.38–32 μg/ml, and according to CLSI breakpoints for slow-growing bacteria (*Haemophilus* sp.), reduced susceptibility (MIC 2–3 μg/ml) in five isolates and probable resistance (MICs≥4 μg/ml) in three strains were demonstrated [21]. We analyzed the mutational profile of *rpoB* to disclose the genetic basis of resistance to rifampicin but none of the identified SNPs have been linked to this phenotype and only synonymous SNPs were detected.

### wgMLST to evaluate *B*. *melitensis* phylogenetic diversity

By using the set of 272 *B*. *melitensis* genome sequences, we were able to generate a curated species-specific wgMLST scheme that enrolls a panel of 2656 targets based on the *B*. *melitensis* 16M reference genome (RefSeq Accession NC_003317 and NC_003318). This wgMLST schema was then applied to investigate phylogenetic relationships between genomes of the 36 PT strains (one was removed from the analysis due to poor quality), to put them in the frame of the worldwide phylogenetic scenario and to disclose potential epidemiological links.

**Fig 1:**
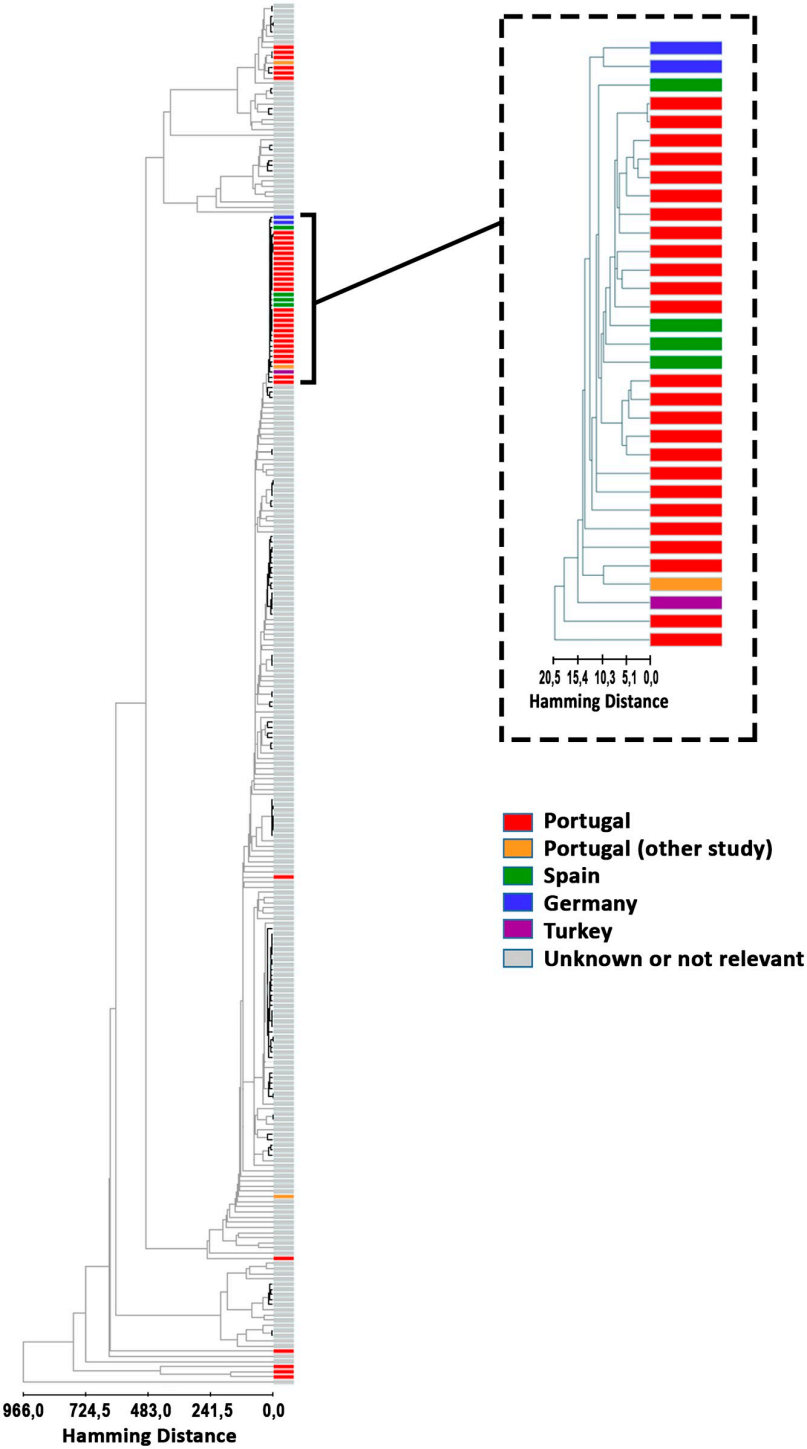
Hierarchical clustering tree, showing the genetic relationship of the 271 *B*. *melitensis* strains collected worldwide, based on a curated species-specific wgMLST scheme that enrolls a panel of 2656 genes. Distances among strains were estimated with Hamming Distance metrics using the single-linkage method. Branch trees representing clusters linked by a genetic relatedness cut-off of 3% are shown in bold black lines. The PT strains are highlighted in red, while strains genetically related to them appear in different colors, concerning the isolation country. The five major genotypes are also displayed above the tree branches. For better visualization purposes, the branch enrolling the largest dataset of PT strains (i.e., genotype II strains) is zoomed-in.

In a first approach, we analyzed the phylogenetic position of PT *B*. *melitensis* strains in a global tree constructed with WGS data from strains collected worldwide (Fig 1). As expected, phylogenetic analysis revealed geographic clustering, according to the previous defined lineages, namely, the American, the West Mediterranean, and the East Mediterranean Region [34]. Although the classification of *Brucella* spp into genotypes is not fully adopted by the microbiologist community, we observed a strains segregation based on the five major genotypes previously described [35]. While genotype I comprises strains from the West Mediterranean lineage, the broader genotype II harbors strains from the East Mediterranean lineage, and genotype III strains from the African continent. On the other hand, genotypes IV and V, which emerged from the same common ancestral derived from genotype III, are assigned to strains from Malta, Portugal and the American Continent. Curiously, despite a few strains cluster in genotype IV clade, the vast majority of the strains isolated in Portugal (25 out of 36) shows up in the clade of genotype II, in particular within sub-genotype IIi [35]. Considering the vast allelic diversity exhibited by all 271 analyzed strains, an empirical genetic relatedness cut-off of 3% was applied to the hierarchical clustering tree in order to have a draft highlight of potential strain clusters, especially those including the 36 PT strains (Fig 1). While PT strains from genotype IV do not seem to have any apparent genetic relatedness with strains isolated in other geographic regions around the world, a different scenario was observed for genotype II. Indeed, the 25 PT strains assigned as belonging to genotype II seemed to exhibit a genetic proximity to strains isolated in Spain, Turkey and to two others isolates from Germany (corresponding to two imported cases with unknown origin).

### Analysis of genetic relatedness among *B*. *melitensis* strains isolated in Portugal

**Fig 2:**
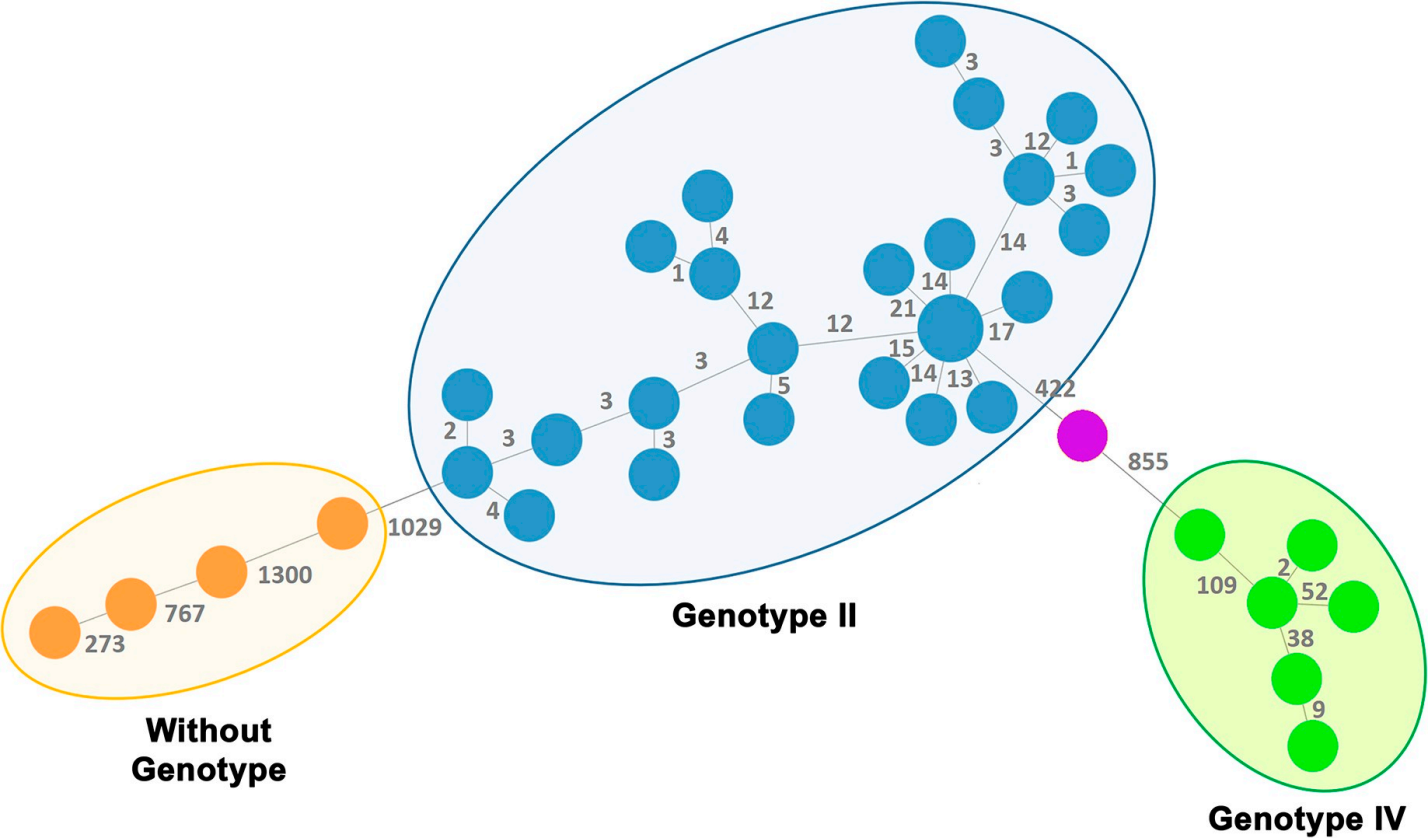
Phylogeny of PT *B*. *melitensis* strains based on a dynamic gene-by-gene approach using a wgMLST schema with 2656 loci. The Minimum spanning tree was constructed using the goeBURST algorithm implemented in the PHYLOViZ Online platform, and is based on the allelic diversity found among the 2191 genes shared by 100% of the 36 PT strains. Filled small circles (nodes) represent unique allelic profiles, and are colored based on the assigned genotype according to Tan *et al* [35]. The size of the circles is proportional to the number of isolates it represents. The numbers in grey on the connecting lines represent the allele differences (AD) between strains.

Considering the allelic diversity found among the 2191 loci that are shared by the 36 PT strains (Fig 2), we were able to zoom-in the scenario of genotype classification described above. It can be observed that strains within each genotype display considerable fewer allelic differences (between one and 21 for genotype II and between two and 109 for genotype IV) than the ones obtained between genotypes or when compared with strains with unassigned genotype, where distances of more than 1000 allelic differences are observed.

**Fig 3:**
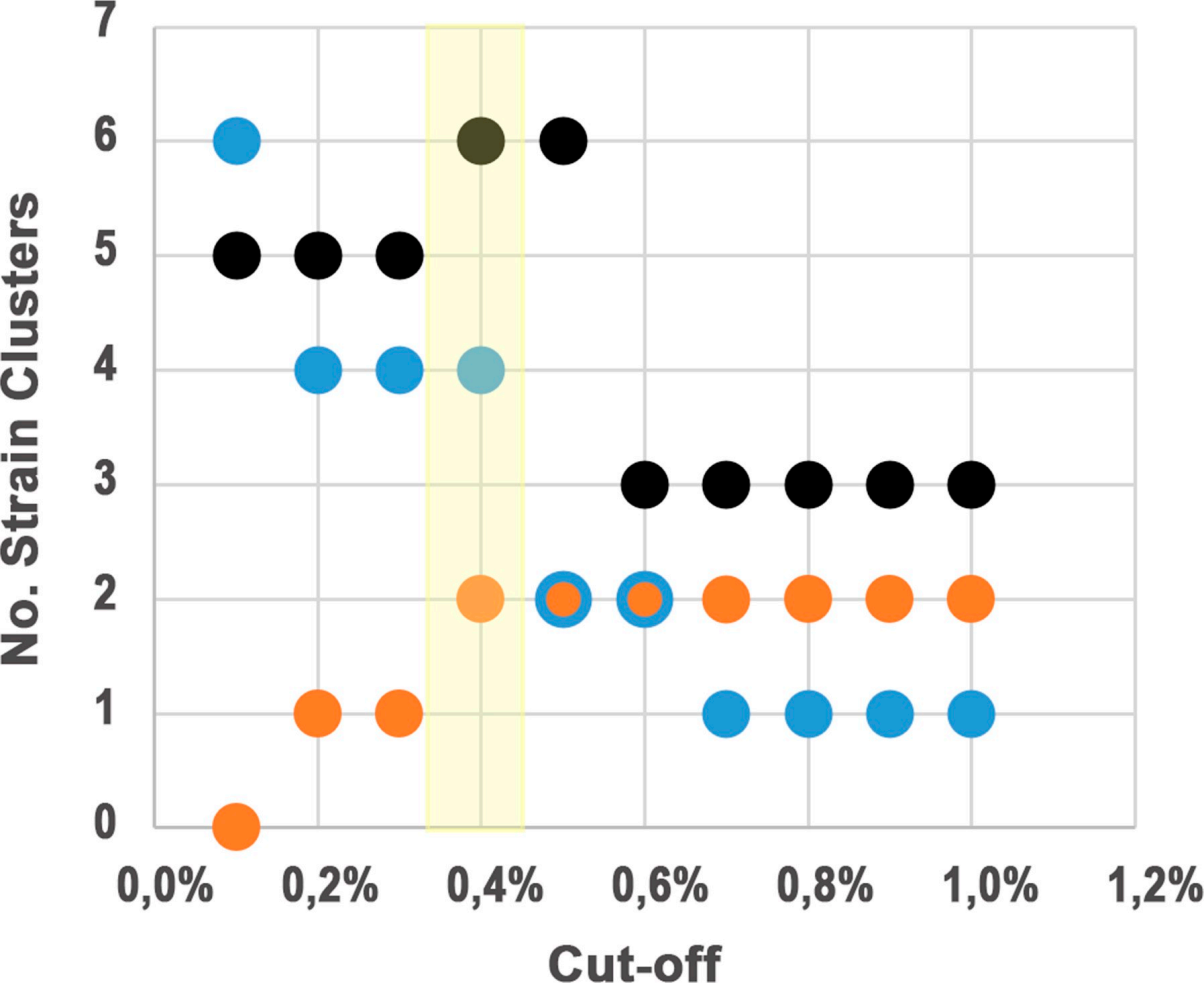
Impact of different allelic distance thresholds on the definition of *B*. *melitensis* strains clusters. The number of clusters are shown for allelic distance cut-offs ranging from 0.1 to 1%. This analysis was done both by genotype and by using all strains. For the present dataset of 36PT strains, the cut-off that maximize the number of clusters identified within each cluster is highlighted in yellow.

**Fig 4:**
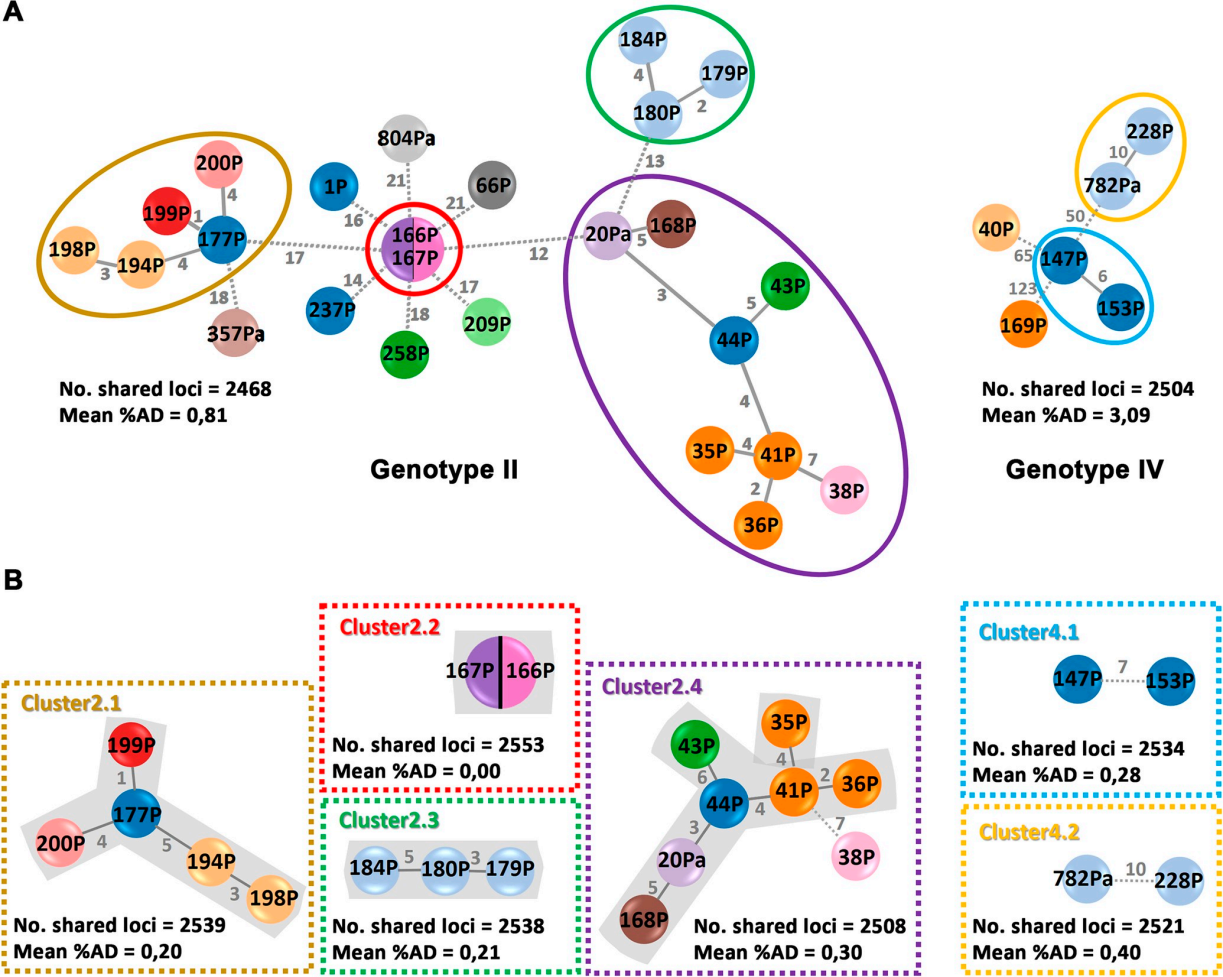
Phylogenetic relationship of PT *B*. *melitensis* strains by genotype based on a dynamic gene-by-gene approach using a wgMLST schema with 2656 loci. (A) For each genotype identified, the initial MST was constructed based on the allelic diversity found among the shared genes between strains (indicated near each tree). Potential clusters defined for fine-scale analysis are surrounded by colored circles and further detailed in panel B. (B) Sub-MST reconstruction based on the maximum number of shared loci (indicated near each tree) between strains forming a putative cluster. For both panels, trees were constructed using the goeBURST algorithm implemented in the PHYLOViZ Online platform. Each filled small circle (node) contains the strain’s designation and represents a unique allelic profile. Nodes are colored according to the geographic region where strains were isolated (see Table 1 for details). The numbers in grey on the connecting lines represent the allele differences (AD) between strains. Within each cluster, strains exhibiting strong genetic relatedness are highlighted in grey and connected by solid lines, while strains with borderline genetic relatedness are connected by dashed lines.

To explore strain sub-sets among our 36 PT strains, two additional MST were generated, one for each genotype. Considering that in *Brucella* spp. there is no defined threshold to identify clusters of genetically related strains with high epidemiology congruence, a conservative step-by-step approach was performed by applying allelic distance cut-offs ranging from 1 to 0.1% to the initial MSTs generated for (i) all 36 (ii) genotype II and (iii) genotype IV strains. We first selected a threshold of 0.4% (that corresponds to ≤11AD) since it allowed to maximize the number of strain sub-sets identified within each genotype (Fig 3). Indeed, after the application of this cut-off to both genotype MSTs, we were able to highlight six genetically related sub-sets of strains, which may harbor a higher probability to have an epidemiological link. In particular, genotype II strains exhibiting ≤10AD remained interconnected in four clusters, and strains from genotype IV with ≤11 AD resulted into two potential related clusters (Fig 4A). Next, for each identified cluster, a sub-MST was generated in order to maximize the number of shared loci among the strain sub-set (Fig 4B), and consequently, to better evaluate the relatedness of strains.

### Evaluation of putative epidemiological links within strain clusters

Regarding both clusters of genotype IV, despite the inexistence of metadata, we cannot discard a possible epidemiological link between the strains. Indeed, while for cluster 4.1, strains were isolated from patients of the same northern village only two months apart, both strains from cluster 4.2 were collected at a city in the center of Portugal (S1 Fig). The later were isolated five years apart (2011 and 2016), where one of them was isolated from a goat whereas the other caused a human infection. Nevertheless, considering the high genetic relatedness and the same isolation locale, one may hypothesize the existence of an epidemiological link between those isolates. Moreover, although in a speculative basis, a low allelic diversity from two isolations of the same strain five years apart would be congruent with an low evolutionary rate of *Brucella* spp.. For genotype II, strains within the cluster 2.3 were from a suspected outbreak in 2014 (based on epidemiological and MLVA-16 data) in a small northern region, due to consumption of raw cheese sold in a local market (Fig 4B and S1 Fig). This outbreak was controlled and the veterinary authorities identified the infected animals (goats) as the likely source of the infection. No culture methods were used so no genome analysis of the strain infecting the goats can be performed at this stage. In an opposite scenario, despite both strains from cluster 2.2 being genetically identical (among the loci analyzed), they were isolated from patients at geographically distant regions from north and center of Portugal (~300km apart) (S1 Fig) in 2011, suggesting that their possible linkage may be related with the ingestion of a product of the same animal origin. Regarding cluster 2.1, *B*. *melitensis* strains were isolated from patients within the Lisbon area between 2014 and 2015, but a possible link was never confirmed. Finally, for cluster 2.4, the largest cluster analyzed, all strains except 44P (for which no information is available) were isolated in the north of the country, where three of them (35P, 36P, 41P) were from the same city and other three (43P, 168P and 38P) were from neighboring small cities (S1 Fig). With exception of 20Pa, which was collected from an animal (goat) in 2002, all strains but 168P (isolated in 2011) were from cases of human brucellosis in 2012. No epidemiological information was available, hampering the determination of the potential infection source.

### Comparison with loci-based and other genome-based approaches

For comparative purposes with the described wgMLST-based approach, in parallel we also evaluated the genetic relatedness among the 36 PT strains with three distinct strategies, namely with: *i)* the traditional MLST schemas based on 9 and 21 loci [32;33]; *ii)* the freely available genus-specific cgMLST schema [16] based on 164 loci (comprising ~6% of the loci panel used in this study); and *iii)* a core-genome SNV-based approach that is also largely used worldwide. In general, similar genotype associations were achieved with the three approaches, but with different discriminatory power. As expected, both MLST-9 and MLST-21 schemas were the least discriminative approaches, not allowing to differentiate strains within each assigned genotype (S2 Fig). Nevertheless, strains within each genotype seemed to hold a characteristic MLST allele profile. On the other hand, a higher strain discriminatory power was achieved with the 164-loci cgMLST, but several unrelated strains still clustered together, especially within genotype II (S3 Fig). Finally, the core-genome SNV-based approach revealed a good strain discriminatory concordance with our wgMLST-based analysis, exhibiting a similar robust tree topology that clearly segregates strains within each assigned genotype (S4A Fig). Indeed, strains from genotype IV displayed a mean of 100.7±7.3 core-SNVs among them but distant a mean of 2005.7±29.3 core-SNVs to genotype II strains. Moreover, within each genotype, the same discrete strain clusters were observed (S4B and S4C Fig), allowing to identify of the same putative epidemiological links.

## Discussion

Brucellosis is a zoonosis that is emerging in some regions of the world; in Portugal, it is an endemic and notifiable disease. Although human cases have been reported throughout the country, it is recognized by the Portuguese Health Authorities that brucellosis cases are clearly underreported, which does not allow consistent analysis of risk factors and the proper evaluation of the impact of this disease on public health. Also, the frequent lack of metadata associated with the isolated strains constitutes a hurdle to the epidemiological research, frequently hampering the identification of the infectious source.

The reference laboratory for human Brucellosis at the Portuguese National Institute of Health receives from the hospital laboratories all human isolates of *B*. *melitensis*, which are typed by MLVA—16 methodology. Aiming at giving a step forward in the *Brucella* spp. surveillance in Portugal, we created a curated ready-to-use species-specific wgMLST scheme enrolling a panel of 2656 targets to perform a retrospective analysis of the genetic relatedness among *B*. *melitensis* strains causing human infection in Portugal. Although two cgMLST schemas were recently developed for *Brucella*, one of them is based on a very small panel of 164 loci [16], and the other runs on a pay-per-use platform (RIDOM SeqSphere) despite involving a wider core gene set of 2704 targets [17].

According to Tan’s classification [35], the isolates from the Portuguese dataset (isolated between 2010 and 2018) essentially clustered in two previously described lineages, namely the East Mediterranean (EM) clade (genotype II) and the Malta and Portugal clade (genotype IV), with few strains falling outside the clades described by Tan and colleagues (Fig 1). The majority of the isolates (25 out of the 36 PT strains) clustered in the Genotype II, in particular within sub-genotype Iii [36], and seem to reveal a genetic proximity to strains isolated in Spain and Turkey (the two close isolates from Germany are imported cases with unknown origin). Such relatedness with strains from Spain is not surprising considering the border free herding, the common traffic of alimentary products from animal origin among these countries as well as the tourism and the free circulation of the population. Other six isolates from the present study clustered in the genotype IV, which correlates well with the extremely common circulation of people between countries where brucellosis is endemic, such as Portugal, Italy and Greece, also sharing similar eating habits. Therefore, the influx of migrations among European countries comes along with raised case counts of an infectious disease.

The implementation of the wgMLST approach allowed us to identify six clusters, where two clusters involve strains from the genotype IV and four clusters involve strains from the genotype II. Despite the absence of complete epidemiological information for most of the cases, our results point to the identification of strong associations between several of them, likely representing missed “outbreaks”. For instance regarding genotype II strains, in the cluster 2.2 two human isolates are genetically “identical”, although they were isolated in different geographical locations. This likely eliminates the hypothesis of the contact with an infected animal but suggests a food origin (e.g., cheese) as the highly likely infectious source. For the cluster 2.3 the genetic analysis shows the correlation with epidemiological data, confirming the outbreak occurred in 2014 in a small northern region that had been identified solely based on epidemiological information (S1 Fig). Considering the unequivocal close genetic relatedness among all strains from the cluster 2.4, their geographic proximity and the existence of an animal infected with likely the “same clone”, ten years before these cases of human brucellosis, we can speculate that this clone is endemic in that region. Thus, it is reasonable to assume that these cases of human brucellosis are likely derived from the consumption of products from infected animals of that specific region.

A tricky issue underlying the application of gene-by-gene approaches, such as the wgMLST reported here, concerns the choice of cut-offs to identify putative genetic linkages and this challenge extends to all microorganisms for which genome-scale approaches are being created. For instance, choosing cut-offs that enable a fine-scale analysis of specific clades of a MST (i.e., enabling a more precise evaluation of the genetic relatedness among the already “most related” strains) modifies its sensitivity, making the exclusion of putative outliers more robust, but may also exclude from the cluster strains with slightly higher genetic differences but with known epidemiological linkage. For instance, by applying a threshold of 0.2% (corresponding to ≤6AD) to each sub-MST (Fig 4B), we were able to consolidate the strong strains’ genetic link within clusters of genotype II, but placed the strain 38P as borderline in cluster 2.4. As no epidemiological information is available for 38P, we cannot assess the accuracy of the chosen threshold for this cluster. In addition, the core-genome SNV-based approach that we used for confirmatory purposes confirmed the putative epidemiological link of strain 38P with the other strains from cluster 2.4, where no SNV were observed to strains 36P and 41P (S4B Fig). Similar genetic relationships among strains were obtained with the SNV-based approach, having observed the same discrete strain clusters previously identified, which strongly supports the robustness and finest strain discriminatory power of the proposed species-specific wgMLST scheme. Considering that gene-by-gene and SNV-based approaches for WGS-based surveillance of *B*. *melitensis* are still at the beginning [37–38; 15–17], the choice for the appropriate cut-offs for cluster definition should be a dynamic process and should always be associated with the existing epidemiological data. On this regard, future studies with large datasets and strong epidemiological data will certainly ensure this achievement.

In conclusion, the application of a WGS-based approach for a retrospective evaluation of the genetic relatedness of all *B*. *melitensis* strains received at the Portuguese reference laboratory between 2010 and 2018 allowed the identification of several highly probable associated cases of brucellosis, where 22 out of the 36 PT strains showed one or multiple genetic linkage with other strains. The putative epidemiological links revealed by this schema were fully supported by the SNV-approach while showed extremely higher discriminatory power than the traditional 9- and 21-loci MLST schemas [32, 33], as well as than the 164 loci based cgMLST previously developed [16]. The implementation of a wgMLST scheme in the reference laboratory constitutes a mark of technological transition for laboratorial surveillance of brucellosis in this country, and will unequivocally facilitate the assessment of ongoing and future outbreaks in order to prevent the transmission spread. It will allow a better understanding of the epidemiology and dynamics of *Brucella* spp. populations and to gather in depth information, which can be used for source tracing in case of outbreaks within animal holdings, zoonotic or foodborne infections.

## Supporting information

S1 Fig Geographic location of the isolated 36 PT *B*. *melitensis* strains. For simplification purposes, the color scheme used to define the clusters is the same as the one presented in Fig 3. Strains belonging to the same putative cluster are connected by the color corresponding to each cluster.
(TIF)

S2 Fig Phylogeny of PT *B*. *melitensis* strains based on the MLST schema based on (A) 9 loci and (B) 21 loci. The Minimum spanning tree was constructed using the goeBURST algorithm implemented in the PHYLOViZ Online platform, and is based on the allelic diversity found among genes of each MLST schema for 35 PT strains. Two PT strains were excluded due to the lower number of alleles called. Circles (nodes) represent unique allelic profiles and are colored based on the predicted ST (outer ring). For comparative purposes nodes were also filled according to the assigned genotype as in Fig 2. The size of the circles is proportional to the number of isolates it represents. The numbers in grey on the connecting lines represent the allele differences (AD) between strains. Asterisks represent strains for which the number of alleles called was 1–2 alleles inferior than the supposed for the MLST schema.
(TIF)

S3 Fig Phylogeny of PT *B*. *melitensis* strains based on a gene-by-gene approach using the genus-specific cgMLST schema with 164 loci. The Minimum spanning tree (MST) was constructed using the goeBURST algorithm implemented in the PHYLOViZ Online platform, and is based on the allelic diversity found among the 164 loci panel [15]. Filled small circles (nodes) represent unique allelic profiles. For comparative purposes with the proposed wgMLST scheme of the present study, nodes are colored similarly to Fig 1 and are grouped based on the assigned genotype according to Tan *et al*. [32]. The numbers in grey on the connecting lines represent the allele differences (AD) between strains.
(TIF)

S4 Fig Phylogenetic relationship of PT *B*. *melitensis* strains by using a core-genome SNV-based approach. For all 36 PT strains (A), global genetic relationships were inferred against the draft genome sequence of Bm-147P (see Methods for details). For all genotype II strains (B), a total of 148 variant sites was identified when mapping to the draft genome sequence of the representative Bm-167P, while for all strains from genotype IV (C), 288 variant sites were found when mapping against Bm-147P. Phylogenies were inferred using the Neighbor-Joining method with the Maximum Composite Likelihood model to compute genetic distances among strains. Bootstrap values (1000 replicates) are shown next to the branch nodes. Potential strain clusters are shown.
(TIF)

S1 Table (DOCX)

S2 Table (DOCX)
