# Supplementary figures and images for: Genome–scale approach to study the genetic relatedness among Brucella melitensis strains
Source: PLoS One. 2020 Mar 9;15(3):e0229863. doi: 10.1371/journal.pone.0229863 (PMC7062273; doi:10.1371/journal.pone.0229863)

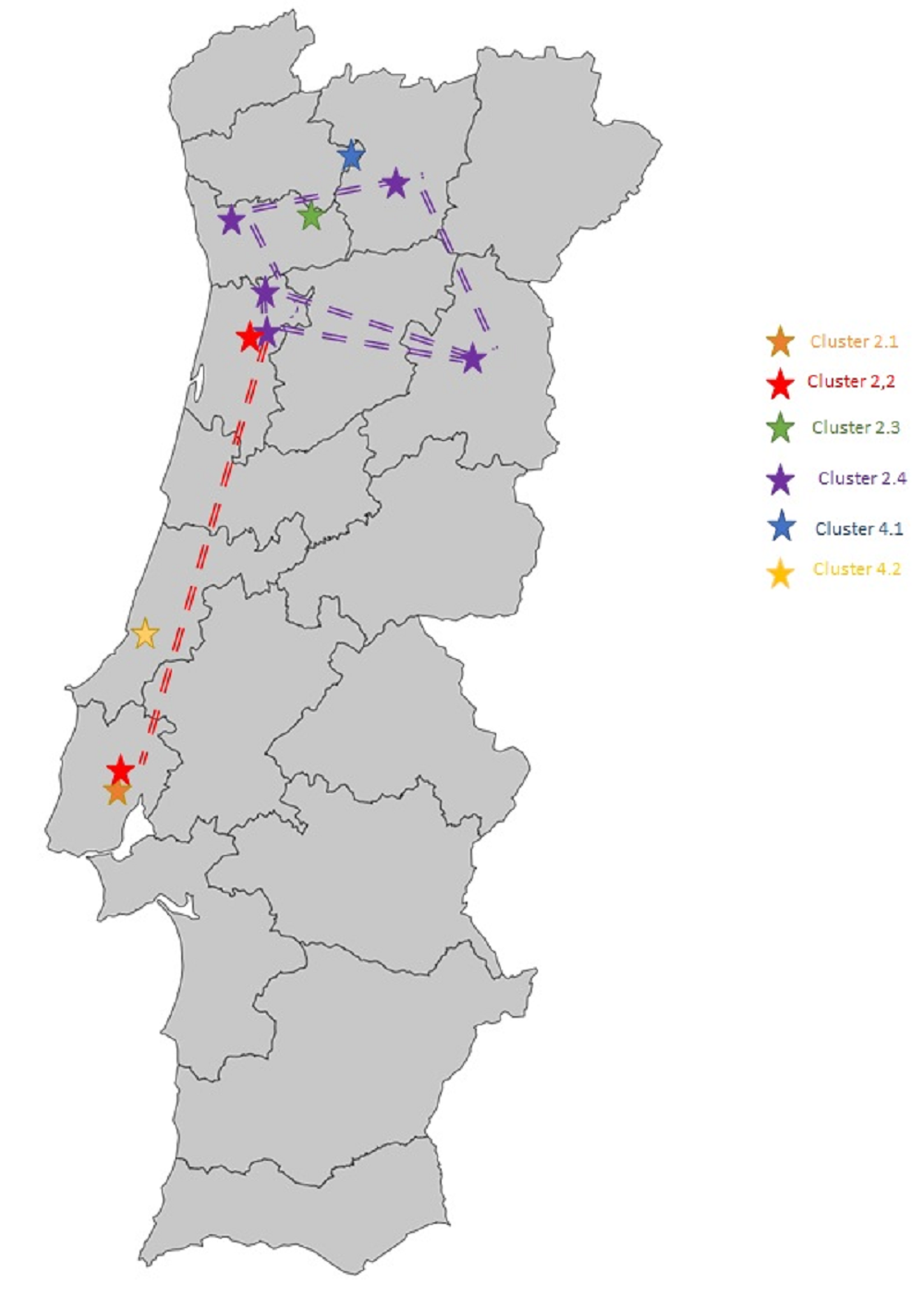

Supplement: S1 Fig — For simplification purposes, the color scheme used to define the clusters is the same as the one presented in Fig 3. Strains belonging to the same putative cluster are connected by the color corresponding to each cluster. (TIF) [file pone.0229863.s001.tif]

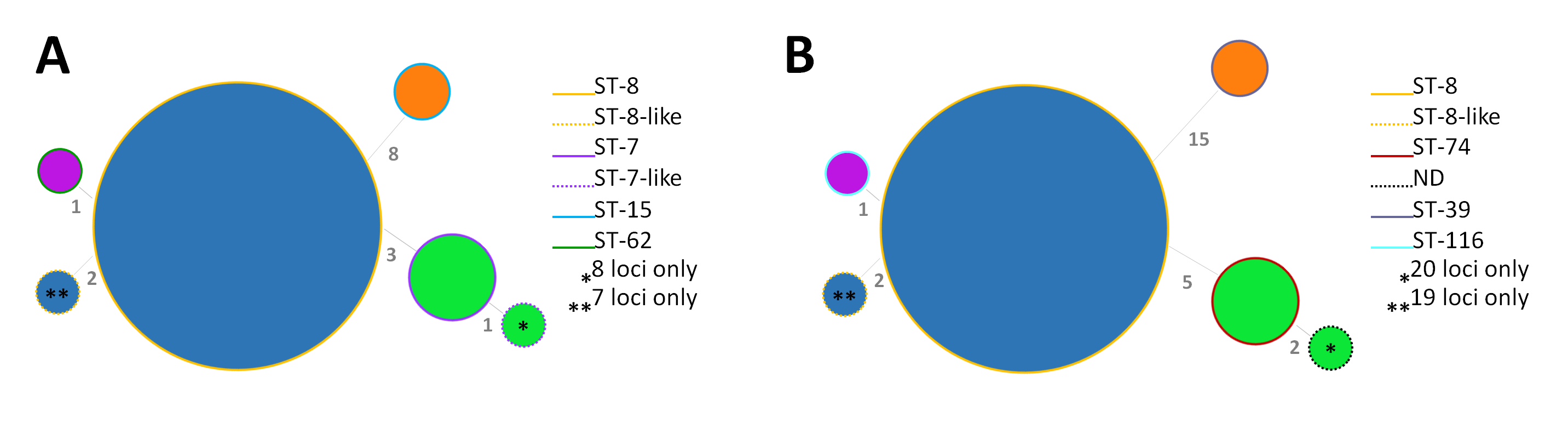

Supplement: S2 Fig — The Minimum spanning tree was constructed using the goeBURST algorithm implemented in the PHYLOViZ Online platform, and is based on the allelic diversity found among genes of each MLST schema for 35 PT strains. Two PT strains were excluded due to the lower number of alleles called. Circles (nodes) represent unique allelic profiles and are colored based on the predicted ST (outer ring). For comparative purposes nodes were also filled according to the assigned genotype as in Fig 2. The size of the circles is proportional to the number of isolates it represents. The numbers in grey on the connecting lines represent the allele differences (AD) between strains. Asterisks represent strains for which the number of alleles called was 1–2 alleles inferior than the supposed for the MLST schema. (TIF) [file pone.0229863.s002.tif]

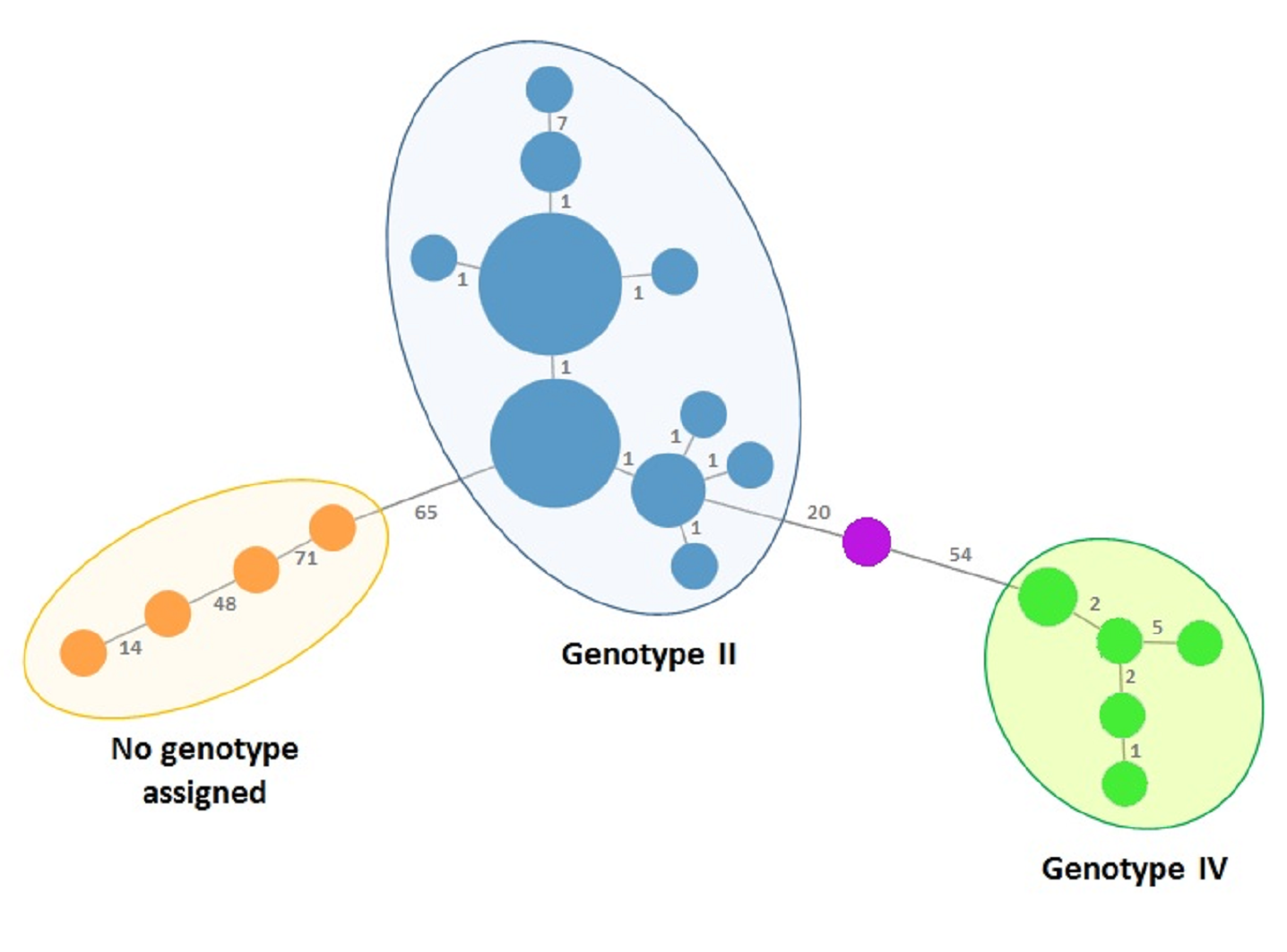

Supplement: S3 Fig — The Minimum spanning tree (MST) was constructed using the goeBURST algorithm implemented in the PHYLOViZ Online platform, and is based on the allelic diversity found among the 164 loci panel [15]. Filled small circles (nodes) represent unique allelic profiles. For comparative purposes with the proposed wgMLST scheme of the present study, nodes are colored similarly to Fig 1 and are grouped based on the assigned genotype according to Tan et al. [32]. The numbers in grey on the connecting lines represent the allele differences (AD) between strains. (TIF) [file pone.0229863.s003.tif]

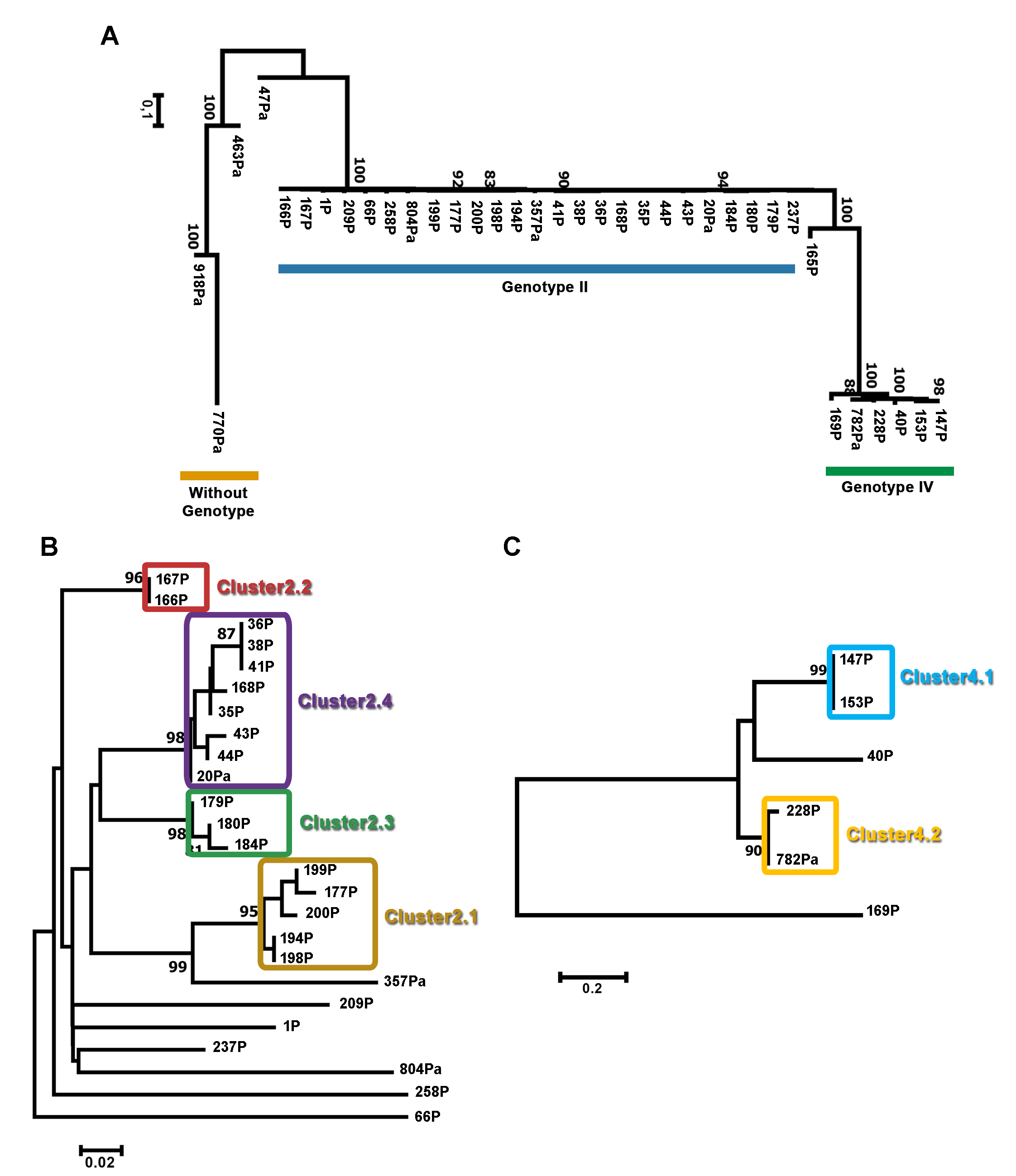

Supplement: S4 Fig — For all 36 PT strains (A), global genetic relationships were inferred against the draft genome sequence of Bm-147P (see Methods for details). For all genotype II strains (B), a total of 148 variant sites was identified when mapping to the draft genome sequence of the representative Bm-167P, while for all strains from genotype IV (C), 288 variant sites were found when mapping against Bm-147P. Phylogenies were inferred using the Neighbor-Joining method with the Maximum Composite Likelihood model to compute genetic distances among strains. Bootstrap values (1000 replicates) are shown next to the branch nodes. Potential strain clusters are shown. (TIF) [file pone.0229863.s004.tif]
