# Supplementary material for: Genome–scale approach to study the genetic relatedness among Brucella melitensis strains
Source: PLoS One. 2020 Mar 9;15(3):e0229863. doi: 10.1371/journal.pone.0229863 (PMC7062273; doi:10.1371/journal.pone.0229863)
Supplement: S1 Table — (DOCX) [file pone.0229863.s005.docx]

Supplementary Table 1 – strain characterization

| **Strain** | **Biosample** | **Run** |
| --- | --- | --- |
| \| 20Pa \| \| --- \| \| 47Pa \| \| 357Pa \| \| 463Pa \| \| 770Pa \| \| 782Pa \| \| 804Pa \| \| 918Pa \| \| 1P \| \| 35P \| \| 36P \| \| 38P \| \| 41P \| \| 43P \| \| 147P \| \| 153P \| \| 165P \| \| 169P \| \| 177P \| \| 179P \| \| 180P \| \| 184P \| \| 194P \| \| 198P \| \| 199P \| \| 200P \| \| 209P \| \| 228P \| \| 237P \| \| 258P \| \| 261P \| \| 40P \| \| 44P \| \| 66P \| \| 166P \| \| 167P \| \| 168P \| | \| ERS2952753 \| \| --- \| \| ERS2952754 \| \| ERS2952755 \| \| ERS2952756 \| \| ERS2952757 \| \| ERS2952758 \| \| ERS2952759 \| \| ERS2952760 \| \| ERS2952761 \| \| ERS2952762 \| \| ERS2952763 \| \| ERS2952764 \| \| ERS2952765 \| \| ERS2952766 \| \| ERS2952767 \| \| ERS2952768 \| \| ERS2952769 \| \| ERS2952770 \| \| ERS2952771 \| \| ERS2952772 \| \| ERS2952773 \| \| ERS2952774 \| \| ERS2952775 \| \| ERS2952776 \| \| ERS2952777 \| \| ERS2952778 \| \| ERS2952779 \| \| ERS2952780 \| \| ERS2952781 \| \| ERS2952782 \| \| ERS2952783 \| \| ERS2952793 \| \| ERS2952794 \| \| ERS2952795 \| \| ERS2952796 \| \| ERS2952797 \| \| ERS2952798 \| | \| ERR2938658 \| \| --- \| \| ERR2938668 \| \| ERR2938653 \| \| ERR2938687 \| \| ERR2938669 \| \| ERR2938670 \| \| ERR2938703 \| \| ERR2938695 \| \| ERR2938677 \| \| ERR2938664 \| \| ERR2938682 \| \| ERR2938646 \| \| ERR2938690 \| \| ERR2938702 \| \| ERR2938647 \| \| ERR2938674 \| \| ERR2938679 \| \| ERR2938684 \| \| ERR2938659 \| \| ERR2938663 \| \| ERR2938676 \| \| ERR2938700 \| \| ERR2938706 \| \| ERR2938705 \| \| ERR2938667 \| \| ERR2938696 \| \| ERR2938704 \| \| ERR2938693 \| \| ERR2938654 \| \| ERR2938680 \| \| ERR2938642 \| \| ERR2938685 \| \| ERR2938656 \| \| ERR2938649 \| \| ERR2938689 \| \| ERR2938645 \| \| ERR2938651 \| |
