# Supplementary material for: Genome–scale approach to study the genetic relatedness among Brucella melitensis strains
Source: PLoS One. 2020 Mar 9;15(3):e0229863. doi: 10.1371/journal.pone.0229863 (PMC7062273; doi:10.1371/journal.pone.0229863)
Supplement: S2 Table — (DOCX) [file pone.0229863.s006.docx]

| Antibiotic agent | MICs Range (µg/ml) of the  *B. melitensis* PT strains | CLSI breakpoints for *Brucella* spp. (µg/ml) | | |
| --- | --- | --- | --- | --- |
|  |  | S ≤ | I = | R≥ |
| Rifampicin* | 0.38 - 12 | 1 | 2 | 4 |
| Doxycyclin | <0.016-1 | 1 | - | - |
| Streptomicyn | 0.5-4 | 16 | - | - |
| Gentamicin | 0.5-2 | 4 | - | - |

Supplementary Table S2. Results of AST testing of *Brucella melitensis*. * CLSI breakpoints for slow-growing bacteria (*Haemophilus* spp.)
